# A neural trait approach to exploring individual differences in social preferences

**DOI:** 10.3389/fnbeh.2014.00458

**Published:** 2015-01-15

**Authors:** Kyle Nash, Lorena R. R. Gianotti, Daria Knoch

**Affiliations:** Division of Social Psychology and Social Neuroscience, Department of Psychology, University of BernBern, Switzerland

**Keywords:** social preferences, individual differences, neural traits, social decision-making, self-control

## Abstract

Research demonstrates that social preferences are characterized by significant individual differences. An important question, often overlooked, is from where do these individual differences originate? And what are the processes that underlie such differences? In this paper, we outline the neural trait approach to uncovering sources of individual differences in social preferences, particularly as evidenced in economic games. We focus on two primary methods—resting-state electroencephalography and structural magnetic resonance imaging (MRI)—used by researchers to quantify task-independent, brain-based characteristics that are stable over time. We review research that has employed these methods to investigate social preferences with an emphasis on a key psychological process in social decision-making; namely, self-control. We then highlight future opportunities for the neural trait approach in cutting-edge decision-making research. Finally, we explore the debate about self-control in social decision-making and the potential role neural trait research could play in this issue.

## Introduction

People display a rich variety of heterogeneity in social preferences. However, individual differences in social preferences are in need of further elucidation, as the field transitions from description to explanation of such heterogeneity (Apicella et al., 2010). Explaining differences in social preferences can deepen our understanding of social decision-making processes and uncover ways to promote more prosocial behaviors. There are key questions to answer. From where do these striking differences in social preferences originate? And once these sources are revealed, what can be inferred about the psychological processes that lead to such individual differences?

We contend that the neural trait approach holds distinct promise in answering these questions. A neural trait can be defined as a quantifiable brain-based characteristic that is stable over time and capable of influencing preferences or behavior. In this article, we detail the neural trait approach to uncovering sources of individual differences in social preferences, particularly as evidenced in economic games. The next section outlines the neural trait approach. The third section highlights contemporary studies across a range of behaviors that have used the neural trait approach to explore heterogeneity in social decision-making. The fourth section outlines future opportunities for the neural trait approach in cutting-edge decision-making research. For example, recent studies sought to uncover divergent motives behind identical behavior through novel combinations of economic games. Further, theorizing about self-control processes in social decision-making is at a crossroads and neural traits may help inform the current debate.

## The neural trait approach

The neural trait approach described here involves indexing brain-based characteristics and examining whether these indices predict behavior in decision-making or processes directly relevant to decision-making. This achieves two respective goals. The first goal is (obviously) to determine sources of behavioral heterogeneity. Decision-making models usually describe aggregate behavior; yet people evidence quite divergent behavior, even in identical situations (Scheres and Sanfey, 2006; Wischniewski et al., 2009; Levallois et al., 2012). Neural traits are well suited to capture this variance in decision-making (Nash and Knoch, 2015). The second goal is to add a level of analysis that supplements and is informed by task-dependent analyses of neural and psychological processes (Berkman and Falk, 2013). Based on prior literature of neural functioning, neural traits can help researchers infer why differences in decision-making may occur. We do note that inferring the psychological mechanism is more suitable when a brain region has been tightly linked to a theoretically relevant function. We also note that because the measurement of neural traits can involve examining the entire brain, the probability of detecting a specious relationship should be controlled for by multiple test correction.

How is a neural trait indexed, then? We hold that an effective neural trait measure should meet two criteria. First, to capture stable differences the neural trait must itself be stable, e.g., the measure should demonstrate high test-retest reliability. Second, the measure should be unique or specific to that individual, or highly capable of disassociating people based on the neural trait measure, much like a neural “fingerprint”.

We focus on two measures that fulfill these criteria. The first is resting-state electroencephalography (resting-state EEG). Measuring resting-state EEG involves recording electrical activity from the brain on the scalp when the participant is at rest to index patterns of brain activity that are not related to any particular task. Power values for different frequency bands are derived from the resting-state EEG in most neural trait research. In fulfillment of the first criterion of neural trait measurement, these frequency-based measures of resting-state EEG activity are relatively stable in the adult brain. They demonstrate test–retest reliabilities of up to 0.8 over a period of 5 years (Dünki et al., 2000; Näpflin et al., 2007), are heritable (estimates ranging from 70–96% heritability; see Zietsch et al., 2007; Smit et al., 2008; van’t Ent et al., 2009; de Geus, 2010), and are unique to the individual (i.e., it is possible to predict who the individual is based on the particular pattern of EEG activity—at up to a 99% recognition rate; Dünki et al., 2000; Näpflin et al., 2007). Thus, resting-state EEG can reliably capture dispositional differences in neural functioning.

Structural magnetic resonance imaging (MRI) of brain anatomy is the second neural trait measure discussed here. Neuroanatomical differences can be quantified by measuring high-resolution images of gray-matter and white-matter. Gray-matter approaches typically quantify cortical and subcortical volume, thickness, or surface area (Ashburner and Friston, 2000), and white-matter approaches quantify features of the neural connections in the brain (Basser, 1995). These approaches are motivated by the assumption that brain anatomy differences reflect functional differences (Boyke et al., 2008; DeYoung et al., 2010). Cortical volume (i.e., the combination of cortical thickness and surface area) is highly stable in the adult brain (Han et al., 2006; DeYoung et al., 2010) and heritable (estimates ranging from 75%–90% heritability; Thompson et al., 2001; Panizzon et al., 2009). White-matter outcome variables have also demonstrated excellent test-retest reliability (Büchel et al., 2004) and are highly heritable (estimates from 75–90% heritability; Chiang et al., 2009). Like resting-state EEG, then, structural MRI measures are ideal for quantifying brain-based individual differences (Kanai and Rees, 2011).

Additionally, both resting-state EEG and structural MRI measures relate to neural and psychological functioning. For example, higher power values in delta, theta, and alpha EEG frequency bands (i.e., slower-wave frequency bands) tend to be associated with decreased cortical activation, whereas higher power values in beta tend to be associated with increased cortical activation (Cook et al., 1998; Laufs et al., 2003; Oakes et al., 2004). Similarly, cortical differences in volume indicate differences in neuronal populations and thereby reflect different functioning or processing capacity in that region (Boyke et al., 2008; DeYoung et al., 2010). Put simply, more cortical volume is generally thought to indicate better functioning. Thus, both neural trait measures are associated with neural functioning, providing an “online” mechanism that can connect stable brain differences to social behavior.

Neural traits also predict functioning in apposite psychological processes. For example, increased resting-state EEG activity in the lateral prefrontal cortex (PFC) is associated with superior response inhibition (Schiller et al., 2013), consistent with the lateral PFC’s reliably demonstrated role in cognitive control (Miller and Cohen, 2001; Aron et al., 2003; Kerns et al., 2004; Knoch et al., 2006; Hare et al., 2009; Cohen and Lieberman, 2010). Increased cortical volume in the same brain region is similarly associated with various measures of cognitive control (Yuan and Raz, 2014). As such, neural traits involving the lateral PFC might reflect differences in cognitive control capacity. However, we wish to mention that research demonstrating the neural and psychological mechanisms that underlie the links between neural traits and social decision-making behaviors is in incipient stages. Below, we highlight several key findings that not only demonstrate how neural traits can explain the sources of heterogeneity in social decision-making but also empirically indicate potential mechanisms.

## Neural trait research: individual differences in social decision-making behavior

Research shows that people demonstrate significant individual differences in cooperative behavior (Kurzban and Houser, 2001), in the rejection of unfair offers (Güth, 1995; Roth, 1995), and in altruistic responding (Andreoni and Miller, 2002). However, gender, economic status, age, and education have difficulty explaining sources of individual differences in social behavior (Camerer, 2003; Henrich et al., 2006). Self-report measures used to explain social preferences also carry certain limitations. Self-report is susceptible to various sources of bias, including socially desirable responding, random responding, and demand characteristics (Edwards, 1957; Nichols and Maner, 2008). Further, completing questionnaires that make certain preferences more salient may alter behavior in a subsequent economic game. Conversely, first completing an economic game may alter responding on subsequent questionnaires.

As an alternative method, neural traits are objectively indexed, free from personal biases and demand characteristics. Moreover, they can be measured remotely from behaviors. Thus, behavioral performance is left unadulterated by the act of completing neural trait measures and vice versa. In this section, we examine research from a diverse sampling of behaviors that fit under the neural trait approach, with a focus on social decision-making behavior.

### Deception

Deception is a social behavior that appears to involve cognitive control as one must deliver a convincing fabrication and simultaneously inhibit the truth (Abe, 2011). Consistent with this, deception reliably engages the lateral PFC (Abe et al., 2007; Sip et al., 2008). However, heterogeneity in the propensity to deceive is poorly understood (Kashy and DePaulo, 1996; Gino and Pierce, 2009). For example, self-report measures of personality traits tend to demonstrate low predictive power of deception (DePaulo, 2004). To address this gap in research using the neural trait approach, Baumgartner et al. (2013a) used a modified trust game to index self-initiated deception and separately measured task-independent resting-state EEG. In the modified trust game, participants played with a partner. Prior to an investment phase, participants signaled to their partner how likely they were to return an investment of money. After the partner transferred money to the participant, they could then make a decision; honor their promise and return the money, or break that trust and keep everything. A whole-brain correlational analysis revealed a surprising finding. In contrast to prior research demonstrating lateral PFC involvement, increased deception was associated with reduced resting-state activation in the anterior insula. The anterior insula is associated with emotional awareness and, in particular, awareness of negative emotions like guilt and shame (Craig, 2009). Baumgartner et al. investigated this by examining participants’ self-reported emotions. They found that increased resting-state activation in the anterior insula also predicted increased negative affect. Thus, these results suggest that people with heightened resting-state insula activity may be predisposed towards honesty because a hyperactive emotional system could make a deceptive act too aversive. More broadly, these results are a compelling example of the power of the neural trait approach. The implementation of a behavior (e.g., deception via the lateral PFC) can be separated from sources of individual differences in that behavior (e.g., heterogeneity in deception is explained by resting-state activity in the anterior insula).

### Intergroup bias in intergroup relations

Increasingly, attention has turned to whether neural traits can explain social preferences in intergroup behavior (Cikara and Van Bavel, 2014), such as those related to egalitarianism and prejudice. Personality measures have been relatively poor predictors of heterogeneity in intergroup bias (Hewstone et al., 2002). Baumgartner et al. (2013b) recognized the need for objective measurement in explaining intergroup bias and thus examined anatomical differences in the brain. They used structural MRI to measure neuroanatomy and measured impartiality with the third-party punishment game. In this game, participants are offered the opportunity to punish both ingroup and outgroup transgressors, though administering such punishment costs money. Results demonstrated that increased dorsomedial PFC volume and (to a lesser extent) temporo-parietal junction (TPJ) volume were associated with increased impartiality. Both the dorsomedial PFC and the TPJ have been strongly linked to perspective-taking processes—recognizing another person’s thoughts, emotions, and goals (Adolphs, 2003; Saxe and Kanwisher, 2003; Van Overwalle, 2009; Young et al., 2010). Based on this, Baumgartner et al. explored perspective-taking as a potential psychological mechanism. They asked participants to rate the degree to which they were able to perspective-take with in-group and out-group members. They found that perspective-taking mediated the association between dorsomedial PFC volume and impartiality. That is, participants with increased dorsomedial PFC volume used perspective-taking more equally for ingroup and outgroup members, and this more equal use of perspective-taking predicted increased impartiality. Together, these results demonstrate that anatomical differences in a distributed perspective-taking network can explain sources of individual differences in egalitarianism. Further, these results demonstrate how neural traits may predict social behavior through a theoretically integral psychological process.

### Indirect reciprocal behavior

People help strangers, even if helping carries a cost and there is no expectation of direct reciprocation (Fehr and Fischbacher, 2003). However, the cost of helping can be defrayed by receiving someone else’s assistance in future interaction (Nowak and Sigmund, 2005). Watanabe et al. (2014) examined heterogeneity in this so-called “indirect reciprocity” by using both functional and structural MRI. Their goal was to tease apart two types of indirect reciprocity; reputation-based (gain a good reputation by reciprocating cooperative behavior) and pay-it-forward (help another after being helped themselves). In terms of the neural trait results, Watanabe et al. (2014) found that increased reputation-based reciprocity was associated with increased precuneus volume and that increased pay-it-forward reciprocity was associated with increased anterior insula volume. This is consistent with the “online” functional results. Increased reputation-based reciprocity was also associated with increased activity in the precuneus and increased pay-it-forward reciprocity was associated with increased activity in the anterior insula. Broadly, this supports the notion that neural traits relate to social behavior through functional differences. More specifically, because the precuneus has been linked to self-centered cognition and the anterior insula has been linked to emotional awareness (Craig, 2009) and affective empathy (Singer et al., 2004), the authors inferred that individual differences in self-centered cognition might explain differences in reputation-based reciprocity and individual differences in affective empathy might explain differences in pay-it-forward reciprocity. Future research should directly probe the degree to which these potential psychological processes mediate the effect of cortical volume in the precuneus and anterior insula on these separate forms of reciprocity.

### Costly punishment behavior

People readily enforce norms, such as the fairness norm, by delivering punishment, even at a personal cost (Fehr and Gächter, 2002). Perhaps because such behavior requires sacrifice, costly punishment behavior is characterized by significant variation across individuals (Herrmann et al., 2008). Findings from the few attempts to explain sources of such behavioral heterogeneity have been mixed, however. Gender, income, wealth, and education have low predictive power that varies strongly according to the idiosyncrasies of the different study designs (Camerer, 2003). On the other hand, up to 40% of variation in costly punishment behavior can be attributed to genetic components (Wallace et al., 2007), yet hinting that stable and objective dispositional differences might be uniquely capable of explaining heterogeneity in costly punishment. Based on this reasoning, Knoch et al. (2010) measured resting-state EEG activity and costly punishment behavior in the ultimatum game. In this game, participants were put in the role of a responder, instructed to reject or accept proposed divisions of real money from a different player (the proposer). A rejection ensures that both parties get no money. Thus the responder can punish the proposer for unfair offers, but at a personal cost. A whole-brain correlational analysis revealed that approximately 50% of the variance in costly punishment was predicted by resting-state activity in the right dorsolateral PFC. It is well established that the lateral PFC is involved in implementing cognitive- and self-control processes (Miller and Cohen, 2001; Aron et al., 2003; Kerns et al., 2004; Knoch et al., 2006; Cohen and Lieberman, 2010). Moreover, costly punishment conflicts with economic self-interest, suggesting that enacting costly punishment requires self-control to overcome the selfish impulse to make money. Based on these considerations, the authors inferred that resting-state activity in the lateral PFC might reflect self-control capacity. Differences in this capacity could thus explain why people differ in costly punishment. This is an explanation that is ripe for direct investigation.

### Altruistic behavior

Defined broadly as behavior that benefits another individual or group at a personal cost to the actor (Camerer, 2003; Fehr and Fischbacher, 2003; Henrich et al., 2006), altruism has been found to have significant, yet difficult to explain individual differences. Andreoni and Miller (2002), for example, found three heterogeneous groups in a modified dictator game; a selfish group, a strategic group, and a fair group. However, characteristics such as sex, wealth, age, and education are poor predictors of individual differences in altruistic behavior (Camerer, 2003; Henrich et al., 2006). Morishima et al. (2012) applied the neural trait approach and used structural MRI to examine if cortical volume might better explain such differences. They expected that anatomical differences in the TPJ and perspective-taking capacity might play a role. Heterogeneity in altruistic behavior was captured from proposals in a dictator game and responses in a reciprocity game. Compared to the ultimatum game, the recipient of the proposal in the dictator game can’t exact punishment, so the proposer acts with impunity. Results showed that increased TPJ volume was associated with increased altruistic behavior (i.e., give money to a partner at a personal cost), particularly when the participant was positioned advantageously to the game partner (Morishima et al., 2012). Further, TPJ volume predicted the cost that each individual was willing to pay for an altruistic act, and this individual-specific cost was associated with functional activation in the TPJ during an altruistic act. Thus, neuroanatomical differences in brain structure can explain differences in altruistic behavior and functional activity in the TPJ during altruistic behavior.

## Future opportunities in the neural trait approach

Research in social decision-making is shifting from simple description to explanation of individual differences (Apicella et al., 2010). We hold, and have hopefully demonstrated in our review, that neural traits can play a significant role in uncovering the sources of these individual differences in decision-making processes and behavior. In the next section, we explore nascent opportunities for the neural trait approach in social decision-making research. First, neural traits can play a role in recent efforts to more precisely determine heterogeneity in behavior by examining behavior across multiple types of games. Second, neural traits can inform the ongoing debate about the role of self-control in social decision-making.

### The neural trait approach and multiple economic games

Cited as a “great success” in decision-making research, economic games have provided researchers with the necessary tools to formalize models that explain significant deviations from self-interested behavior and the prototype of *Homo economicus* (see Fehr and Krajbich, 2013). Recent findings highlight another emerging research opportunity. The meaning or motives behind certain behaviors in economic games have been hotly contested. A single economic game played with one-shot interactions can reveal individual differences in cooperative behavior. But why are people cooperative in that game? Is there only one motive driving behavior for all people? Or can people be cooperative for different reasons? Whereas multiple economic games have been employed primarily to examine behavioral consistency (Yamagishi et al., 2013; Peysakhovich et al., 2014), administering multiple games to the same participant can also allow the researcher to isolate within-person motives to more precisely demonstrate the nature of these social decisions (Brañas-Garza et al., 2014). Research that used this methodology has revealed that the same behavior (e.g., cooperative behavior) is often multi-determined and can be attributed to entirely different motives.

As an example of this multiple game methodology, Yamagishi et al. (2012) tested whether costly punishment in the UG reflects a preference for fairness. They found that this behavior did not correlate with fairness-related behavior in prisoner’s dilemma, trust, and dictator games. Yamagishi et al. (2012) surmised that costly punishment was driven by social-status concerns. Brañas-Garza et al. (2014) also used multiple games to examine whether costly punishment in the UG reflects a benevolent tit-for-tat strategy (i.e., fairness), as is commonly assumed, or a spiteful riposte to an insulting offer. These researchers focused on people who rejected unfair offers in the UG and examined their behavior as proposer in the DG. Two groups emerged. One group proposed fair offers and another group proposed unfair offers in the DG (Brañas-Garza et al., 2014). For this latter group, purely selfish monetary gain is not the driving motive, hence the rejection of money, nor is fairness, hence the unfair proposal in the DG. Rather, these people appear to reject unfair offers purely out of spite. Thus, evidence for both fairness and spitefulness motives were found, though amongst separate groups of people.

The use of multiple economic games to characterize heterogeneity in decision-making has even been used recently to isolate the elusive *Homo economicus*—a model of behavior in which the person maximizes personal gain with no regard for others. Yamagishi et al. (2014) had participants play as second mover in the prisoner’s dilemma game (PDG) and proposer in the DG, behaviors that should cleanly reveal either selfish or prosocial preferences. They focused on two selfish groups. One group, aptly termed Homo economicus (comprising 7% of the sample), always kept their money in the PDG and DG. Another group, termed quasi-Homo economicus (comprising 8.7% of the sample), were nearly as selfish, though they gave money to their playing partner a small fraction of the time. Further, based on personality profiles, the authors reasoned that the Homo economicus group appeared more rational, whereas the quasi-Homo economicus group appeared intuitive (Yamagishi et al., 2014).

Thus, administering multiple games to the same individuals can better describe individual differences, particularly amongst people who evidence the same behavior in a single game. However, the sources of such differences remain unexplained. Neural traits are ideally positioned to explain these more fine-grained motives and preferences. In studies that administer several games, it is particularly critical that neural traits can be measured separately and objectively from social behavior, controlling for bias and demand characteristics that could adversely impact multiple instances of behaviors. For example, what are the sources of individual differences between Homo economicus and quasi-Homo economicus groups? These authors speculated that these groups differed in rationality and cognitive control capacity. The neural trait approach could probe whether Homo economicus and quasi-Homo economicus demonstrate differences in resting-state activity or structure in the lateral PFC, importantly, without adulterating the multiple instances of behaviors.

### Self-control in social decision-making

Self-control is commonly implicated in many social behaviors. Self-control is the process in which thoughts, emotions, or prepotent responses are inhibited to efficiently enact a focal goal. Self-control is thus used to resolve conflicts between competing motives, and most economic games invoke such a conflict as prosocial behaviors often carry pecuniary sacrifice. However, there is an ongoing debate about which kind of behavior is prepotent—selfish or prosocial behavior—and, as a result, which behavior requires self-control to implement. On the one hand, the classic assumption has been that selfish desires are automatic or primary impulses and prosocial desires are secondary, requiring self-control to implement. This assumption has been reliably affirmed in past work (van’t Wout et al., 2005; Knoch et al., 2006, 2010; for a review, see Knoch and Nash, 2015) and contemporary evidence continues to mount in favor of prepotent selfishness. For example, Strang et al. (2014) experimentally decreased left and right lateral PFC activity in a within-subjects design and had participants act as the proposer in two conditions of the dictator game; a condition with no punishment and a condition in which the responder may punish the proposer (similar to an ultimatum game). Results showed that only disruption of the right lateral PFC caused a reduction in the strategic shift from lower offers in the no-punishment condition to higher offers in the punishment condition. In a separate study, Ruff et al. (2013) similarly had participants play as the proposer in a dictator and ultimatum game and manipulated right lateral PFC activity, though activity was either increased or decreased in a between-subjects design. Results showed that increased right lateral PFC activity caused an increased strategic shift in offers in the ultimatum game from the dictator game, whereas decreased right lateral PFC-activity caused a reduction in this strategic shift, as compared to a sham activation control group. Together, these results support the idea that the lateral PFC plays a causal role in the implementation of norm compliant behavior and, more appositely, support the view that prosocial behavior requires self-control.

On the other hand, researchers have found evidence contrary to this classic assumption—i.e., prosocial impulses are primary and require no self-control to act upon. For example, Rand et al. (2012) found that more automatic or intuitive processing led participants to behave more prosocially across a range of social decisions (Rand et al., 2012). An fMRI study demonstrated that accepting, and not rejecting, unfair offers in the ultimatum game involves the lateral PFC, suggesting that the lateral PFC was involved in controlling automatic prosocial impulses to enact the selfish choice of keeping money (Tabibnia et al., 2008). Finally, a number of studies have found that prosocial behavior involves reward-related brain regions and does not involve the self-control-related lateral PFC (for a review, see Zaki and Mitchell, 2013). These studies thus evidence that prosocial behaviors can also be “impulsive” or intuitive.

These competing results demonstrate that the current debate could benefit from an integrative perspective. It appears that there is no one impulse that is universally prepotent or “default”; rather prosocial and selfish desires can both be prepotent. What determines a person’s prepotent impulse is personality and the environment. This position aligns with classic psychological principles (e.g., Lewin (1946) famously elegant equation, B[behavior] = P[personality] × E[environment]) and hews closer to Occam’s razor as the need to posit some universal, chronically prepotent impulse is obviated. Further, this view aligns well with contemporary theorizing on prosocial behavior. For example, Declerck et al. (2013) outlined a neuropsychological model of cooperation to demonstrate that selfish and prosocial “rationales” for cooperative behavior need not be contradictory if we recognize that the brain can implement both types of cooperation. In this model, other-regarding and self-regarding preferences, identified as “individual inclinations”, interact with “contextual influences” (i.e., “E”, situational or cultural factors; Herrmann et al., 2008; Izuma, 2012) to moderate neural and psychological processes in cooperative decision-making and behavior. To enact cooperative behavior, people with self-regarding preferences require extrinsic incentives for context-specific cooperation. Overcoming selfish motives and implementing prosocial behavior, for those with self-regarding preferences, requires cognitive control via the lateral PFC (for supportive experimental evidence, see Emonds et al., 2011).

Neural traits could figure prominently in investigating a neuropsychological model of social preferences and contextual influences. For example, one could first measure neural traits and then measure neural activation during the social behavior. “Offline” psychological capacity, as revealed by the neural trait, should thus relate to “online” functioning during decision-making processes or behavior, perhaps moderated by contextual influences. As an illustration of this, recall the study described above in which resting-state activation in the lateral PFC helped explain individual differences in costly punishment behavior (Knoch et al., 2010). Theoretically, resting-state activation in the lateral PFC could be positively associated with online activation in the lateral PFC and self-control during costly punishment behavior, particularly in the presence of extrinsic incentives for context-specific cooperation. On the other hand, it is also possible that certain neural traits are associated with more efficient use of certain brain areas or systems and could be related to *reduced* rather than increased online activation in respective brain regions. Such findings would cast an entirely new light on research involving task-dependent activity. These are intriguing open questions well-suited to the neural trait approach.

## Conclusion

We have outlined the ways in which neural traits may meet the demands of a changing field of research currently shifting from description towards explanation. Neural traits offer an objective, stable measure to uncover sources of heterogeneity in social preferences. In closing, we first wish to highlight a limitation. Researchers should be aware that in relating decision-making behavior to certain brain areas, there is an inclination to “guess” at the psychological process that mediates the brain—behavior link. Indeed, this potential was often discussed in this paper as an advantage of the approach. To be clear, however, inferring the psychological mechanism is more appropriate when a brain region has been tightly linked to specific, germane functioning. We reviewed several studies that attempted to provide mechanistic evidence. Recall the impartiality research in which Baumgartner et al. (2013b) found that perspective-taking processes mediated the link between dorsomedial PFC volume and impartiality. These results supported their inference that people with increased volume in the dorsomedial PFC had improved perspective-taking abilities and are able to employ this capacity more equally for in and outgroup members, leading to reduced bias. Future neural trait research should similarly probe both neural and psychological mechanisms that underlie the links between neural traits and individual differences in social decision-making behaviors.

We close by noting exciting potential applications. Though neural traits are highly stable across time, they should not be immutable. Indeed, certain techniques and/or experiences impart enduring changes to neural structures and processes. For example, techniques such as neurofeedback, meditation, or repeated practice of skills have the capacity to increase cortical volume or cortical baseline activity in specific brain regions (e.g., Lazar et al., 2005; Takeuchi et al., 2010; Ghaziri et al., 2013). Thus, targeted training manipulations of specific neural traits related to social preferences might impart stable changes to social preferences and self-control capacity. Finally, we focused on stable neural traits in healthy adults. A rich area of investigation could consider neural traits vis-à-vis a fuller perspective that included genetics, development, hormones, physiology, etc. (e.g., Burnham, 2007; Eisenegger et al., 2011). For example, the neural trait approach can occupy a unique position in genetic research on social preferences and decision-making. The *intermediate phenotype model* views neural mechanisms as the intermediate stage through which genes direct behavior (Meyer-Lindenberg and Weinberger, 2006). Neural trait measures are ideal intermediate phenotypes given that intermediate phenotypes are defined as stable and heritable (e.g., Gianotti et al., 2012). Researchers could thus probe unique gene → trait → relations in social preferences and decision-making.

## Conflict of interest statement

The authors declare that the research was conducted in the absence of any commercial or financial relationships that could be construed as a potential conflict of interest.
